# Highly compact refractive index sensor based on stripe waveguides for lab-on-a-chip sensing applications

**DOI:** 10.3762/bjnano.7.66

**Published:** 2016-05-25

**Authors:** Chamanei Perera, Kristy Vernon, Elliot Cheng, Juna Sathian, Esa Jaatinen, Timothy Davis

**Affiliations:** 1 Science and Engineering Faculty, Queensland University of Technology, Brisbane 4001, Queensland, Australia; 2 Australian National Nanofabrication Facility QLD node, University of Queensland, St Lucia 4072, Queensland, Australia; 3 Department of Materials, Imperial College, London SW7 2AZ, UK; 4 School of Physics, University of Melbourne, Parkville 3010, Victoria, Australia

**Keywords:** interferometer, sensing, surface plasmons, waveguides

## Abstract

In this paper we report the design and experimental realisation of a novel refractive index sensor based on coupling between three nanoscale stripe waveguides. The sensor is highly compact and designed to operate at a single wavelength. We demonstrate that the sensor exhibits linear response with a resolution of 6 × 10^−4^ RIU (refractive index unit) for a change in relative output intensity of 1%. Authors expect that the outcome of this paper will prove beneficial in highly compact, label-free and highly sensitive refractive index analysis.

## Introduction

Plasmons are coherent oscillations of free electrons existing on metal dielectric interfaces and are highly sensitive to the surrounding dielectric environment. This unique property is incredibly useful in sensing applications. Mach–Zehnder (MZ) interferometry [1–5], surface enhanced Raman spectroscopy (SERS) [6–9], ring resonators [10] and surface plasmon resonance (SPR) [11–13] are widely used techniques utilising plasmons to measure refractive index changes.

SPR is the most widely used sensing technique. It provides high sensitivity with label-free detection. Most commercially available SPR sensors employ a prism configuration to excite SPs on a metal surface which makes the device too large for lab-on-a-chip applications [3] and requires precise alignments [12,14].

Nano-plasmonic sensors utilising metallic nanostructures can be used to overcome these limitations. In addition, they allow miniaturisation of the overall device size as well as improving the ease of excitation. The plasmonic Mach–Zehnder interferometer (MZI) is one such alternative passive nano-optical device used in refractive index sensing applications [3,5,15–16]. In physics, a MZI is a device used to determine the relative phase shift variations between two collimated beams derived from splitting light from the same single source [17]. These nano-sized MZI based sensors typically have a sensitivity 10–100 times less than that of the commercially available SPR-prism based sensors, but have the advantage of being more compact and having sufficient sensitivity for being used in some lab-on-a-chip applications [3].

Batoli et al. have designed a MZI integrated on a microfluidic platform consisting of two parallel nanoslits in a metal film coated on a glass substrate [3,18]. One slit was used to scatter white light into SP modes on a metal/fluidic interface (sample) on top and a metal/glass interface (reference) at the bottom. Launched SPs on the top and bottom surfaces travelled along the interfaces toward the other slit. These two SP modes interfered with each other and modulated the far-field scattering at this slit. Far-field intensity depends on the phase shift between two SP modes. The resolution of this MZI was 1.5 × 10^−4^ RIU while the minimum size of the device had a lateral dimension of 23 µm.

Pacifici at el. also developed an MZI consisting of two grooves separated by a slit [5]. White light was directed onto the grooves and excited multiple SPs with different frequencies. Counter propagating SP waves interfered with the incident light at the slit location causing modification of the light intensity transmitting through the slit. The intensity of the transmitted light carries information about the near-field interaction of the plasmon with the dielectric environment. The resolution of this sensor was 3 × 10^−7^ RIU.

All of the interferometric designs described require exciting a SP on a metal slab with a surface defect (such as a groove or slit) and then analysing the resulting interference pattern transmitted through a slit. These sensors enable excitation using white light beams and generate interference patterns from multi-frequency SPs. Most of the presented MZI designs are at least micron sized and it is difficult to analyse two analytes at once. As an example, the sensor proposed by Pacifi et al. needs to run separately for reference and sample solutions to allow analysis [5].

MZIs can be designed to be wavelength specific and more compact using waveguide structures. Vernon et al. proposed a compact interferometer design using stripe waveguide coupling to measure the change in the refractive index of a sample using the change in the output intensity [19]. The stripe waveguides in the proposed design were supporting long-range surface plasmon polaritons (LRSPPs). The design consisted of four stripes in total. Light coupled into the sensor using the input arm with the generated LRSPP travelling along the input arm before coupling into the two outer reference and sample arms. Waves propagating along the two outer arms then evanescently coupled into the output arm. The presence of a sample in the sample arm caused a phase change in the wave travelling in the sample arm. This phase shift was then detected by the change in the output intensity at the output arm.

**Figure 1:**
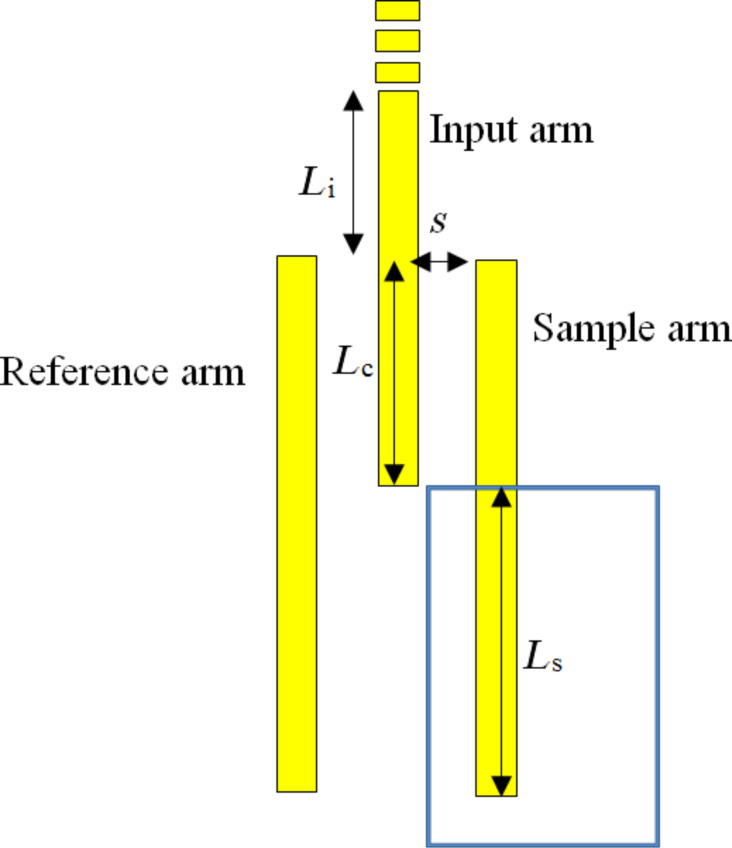
Schematic diagram of the proposed sensor. The sample window is shown as the blue rectangle. *L*_i_ is the length of the input waveguide before coupling with the outer arms, *L*_c_ is the coupling length, *L*_s_ is the sample length and *s* is the separation between inner arm and outer arms.

In this paper, we report the realisation of a refractive index sensor based on stripe waveguide coupling (see Figure 1). The structure is similar to the structure proposed by Vernon et al. [19] but without the output coupling arm, thus reducing the overall size of the device. The sample window was etched on top of the sample arm via a second electron beam lithography (EBL) process, and was used to place sample solution on top of the sample arm. The presence of the sample changes the wavenumber of the propagating plasmon mode hence changes the output intensity at the end of the sample arm. The sample arm and reference arms must be separated from the input arm sufficiently so that the propagating modes do not couple between the sample and reference arm, only between the input arm and the outer arms. The length of the waveguides must also be chosen to ensure there is full transfer of energy between the input and outer arms (i.e., the lengths of the arms are determined by the coupling length of the system). A schematic of the interferometer design is given in Figure 1.

## Results and Discussion

### Theory

For the MZI based on stripe waveguides, the waveguide is required to support a single LRSPP mode (ss_b_^0^) at 633 nm excitation wavelength. Previously it has been reported [20] that this can be achieved with a silver stripe waveguide of width 750 nm and thickness 30 nm. The overall sensor design consists of three identical stripe waveguides labelled as input arm, reference arm and sample arm (Figure 1). The ss_b_^0^ mode of the input arm can be excited via end-fire excitation or grating coupling. This mode propagates along the input arm until its evanescent field starts to interact with the two outer arms.

**Figure 2:**
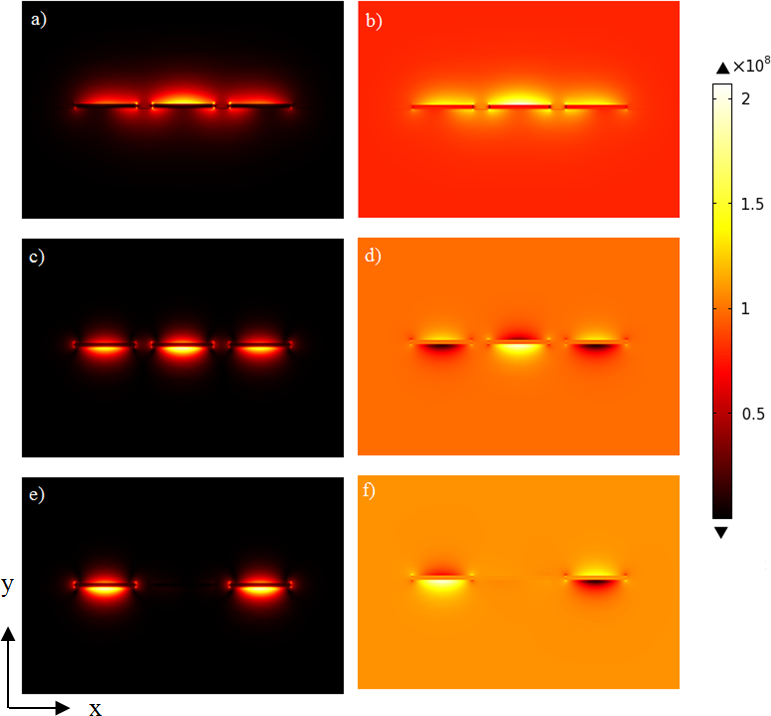
(a), (c) and (e): Magnitude of the electric field distribution of the eigenmodes 1, 2 and 3. (b), (d) and (f): The *y*-component of the electric field for eigenmodes 1, 2 and 3. Stripes are made from silver, 750 nm wide, 30 nm thick, and separated by 200 nm on ITO coated glass substrate with PMMA cladding for an excitation wavelength of 633 nm. These simulations were done using COMSOL Multiphysics.

*L*_i_ is the length of the input waveguide before coupling with the outer arms, *L*_c_ is the coupling length and *L*_s_ is the sample length. The sample arm and reference arm are separated from the input arm by a separation distance *s*, and are uncoupled to each other. This situation can produce only three eigenmodes as shown in Figure 2. For more detail on these eigenmodes and COMSOL simulations refer to [19].

The total field of the coupled system is a linear combination of these three eigenmodes and can be derived using the Haus and Fonstad approach [21]. Refer to Supporting Information File 1 for more details. The parameters of the proposed design are separation distance (*s*) 200 nm, *L*_i_ = 5 μm, *L*_c_ = 29 μm and *L*_s_ = 20 μm.

### Intensity analysis

We are interested in the output intensity difference at the sample arm and the reference arm. The corresponding *z* at this point is

[1]
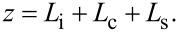


The electric field of the reference arm at this point is

[2]
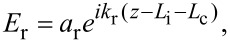


where *k*_r_ is the wavenumber of the mode travelling along the reference arm. The intensity from the reference arm (i.e., at *z* = *L*_i_ + *L*_c_ + *L*_s_) can be determined using

[3]
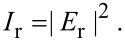


The wavenumber of the propagating mode on the stripe waveguide changes due to the presence of a sample. The electric field of the sample arm at *z* = *L*_i_ + *L*_c_ + *L*_s_ is given by

[4]
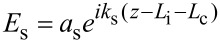


and the corresponding intensity can be found using

[5]
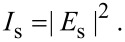


To determine the sensitivity, we analyse the intensity difference between reference arm and sample arm due to a small change in the sample refractive index.

### Fabrication

**Figure 3:**
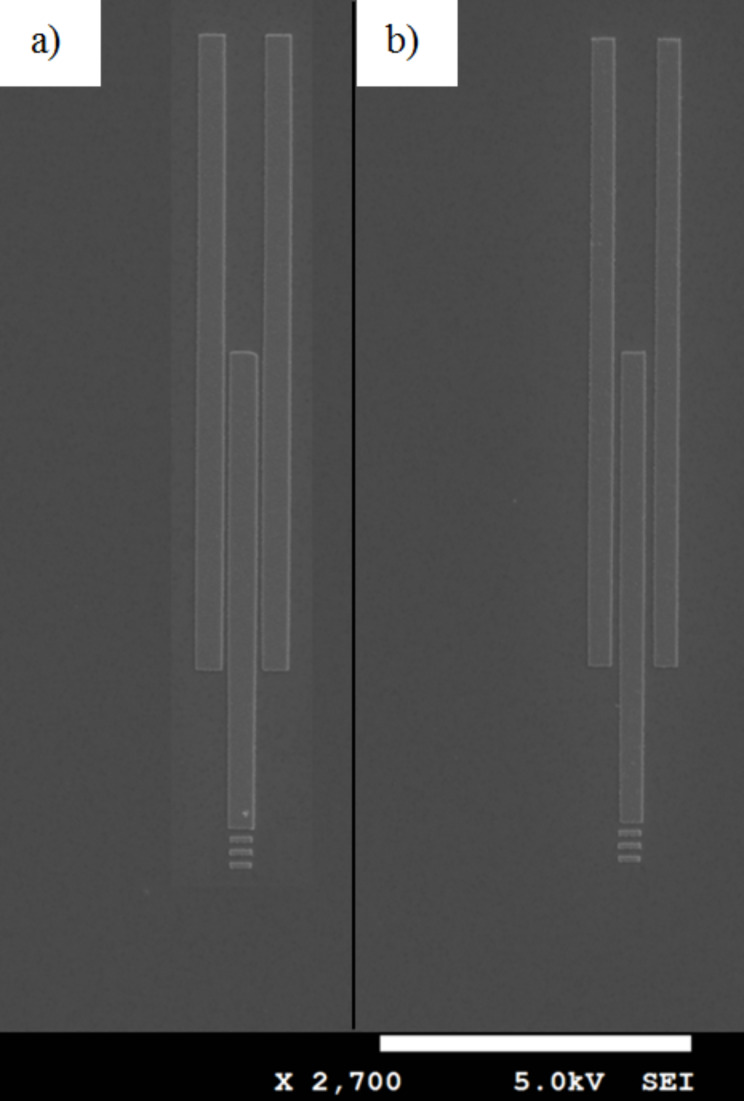
SEM images of interferometers with gap separation a) 200 nm and b) 300 nm. The scale bar in the figure represents 10 µm.

The refractive index sensor (RI sensor) with a sample window on top of the sample arm was fabricated using a multi-step e-beam lithography (EBL) technique. First, the RI sensor designs and alignment marks were patterned on a 300 nm bilayer PMMA resist (950 k A4 / 495 k A4 PMMA resist from Microchem GmbH) using electron beam lithography (JEOL-7800 FE-SEM with Raith Quantum Elphy) with a beam current of ≈75 pA and 20 kV acceleration voltage under optimal dose of 280 μC/cm^2^. The patterned PMMA bilayer was then developed for 30 seconds in MIBK/IPA 1:3 solution. 30 nm Ag was evaporated on the developed sample using the PVD 75 e-beam evaporator under a slow rate of 0.1 angstrom per second. A scanning electron microscope image was obtained (Figure 3) which shows the successfully survived sensor designs after lift-off in acetone bath. Second, a 300 nm bilayer PMMA resist was spincoated on top of the structures to pattern the sample window on top of the sample arm. Then, the sample windows were patterned in the second EBL step. Finally, the patterned bilayer was developed in the developer solution for 30 seconds.

Lengths of the input guide, reference arm and sample arm were 15 µm, 25 µm and 25 µm respectively. The design was tested for two separation distances of 200 nm and 300 nm as shown in Figure 3.

### Testing

**Figure 4:**
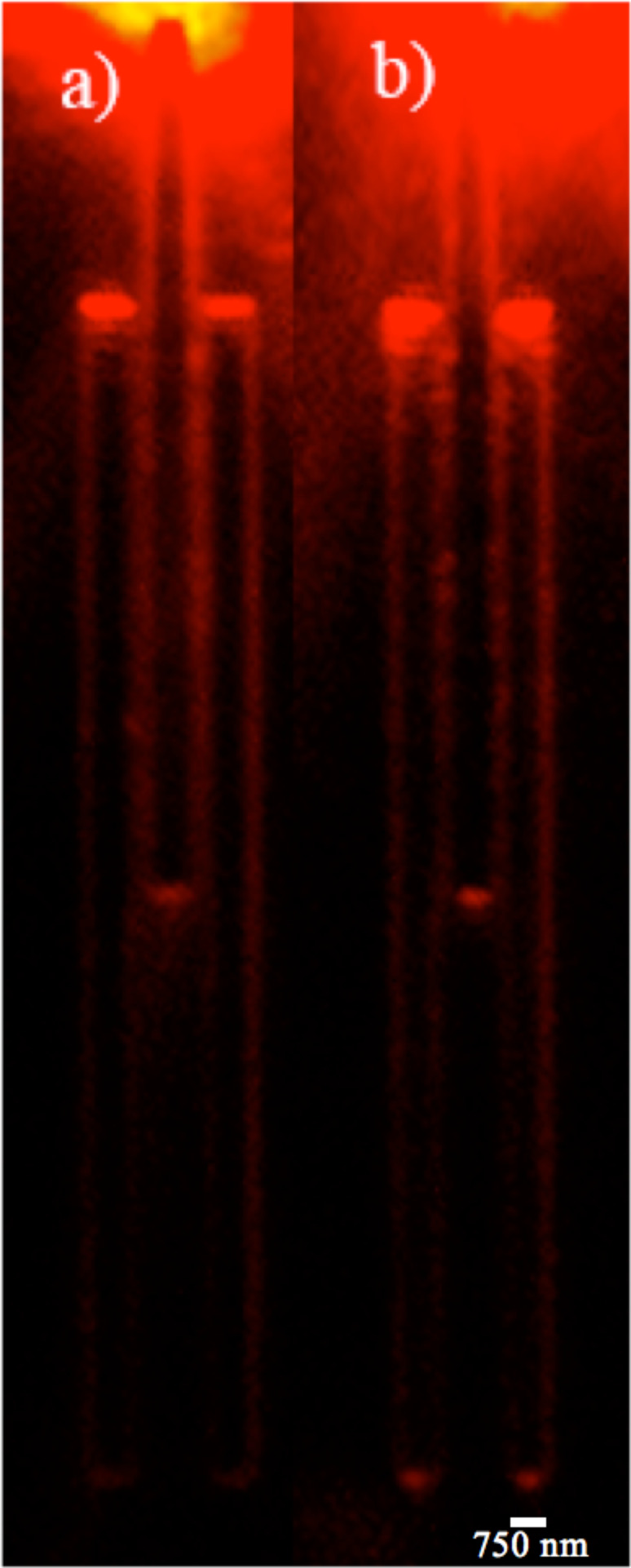
QD luminescence images of interferometers with 300 nm gap and 200 nm gap when excited via input arm with a 633 nm laser.

A grating structure was placed at the input end to increase the energy transfer of incoming light into the input waveguide [22]. The gratings were excited through an inverted microscope set-up using a 633 nm wavelength laser, further details are provided in [22]. The plasmon propagation and coupling in the RI sensor was imaged using CdSe quantum dots (QDs) with emission wavelength at 655 nm (from Invitrogen Cat. No. Q21321MP). An optimum QD spacer layer thickness of 18 nm SiO_2_ was selected [23]. The sensor designs with separations between two outer arms of 300 nm (Figure 4a) and 200 nm (Figure 4b) were observed under QD luminescence.

We observed that plasmon outcoupling intensity was higher when the outer waveguide separation is lower. Based on the experimental results obtained from the QD images we chose to proceed with the sensor with 200 nm outer arm separation.

**Figure 5:**
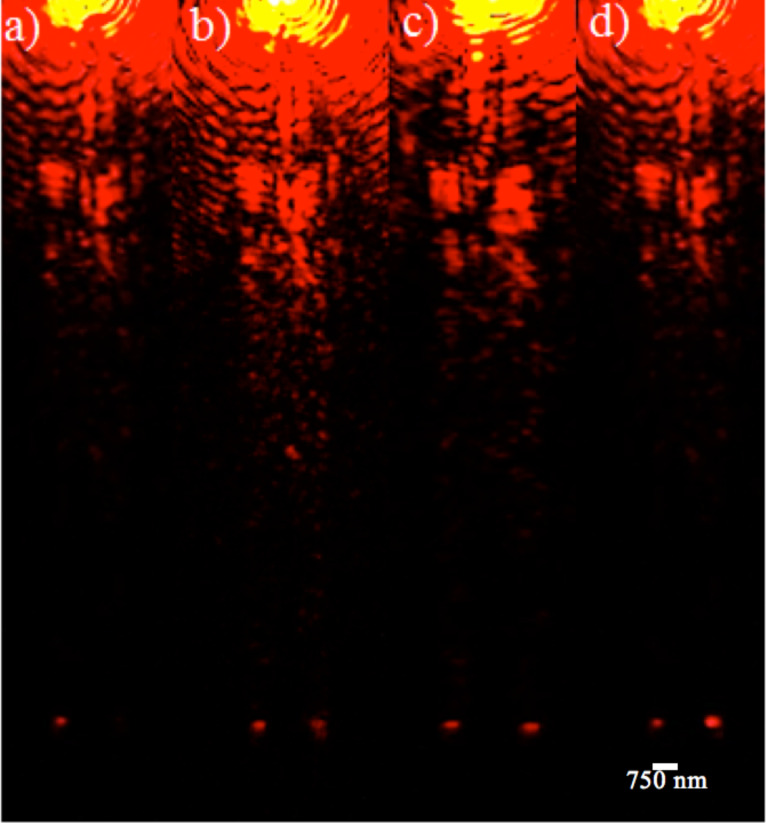
Plasmonic RI sensor. Right outer arm (reference arm – covered with PMMA), left outer arm (sample arm – covered with 0.5% sucrose in DI water). Rightmost to left are series of CCD images obtained for 0%, 10%, 80% sucrose in DI water and index matching oil.

An input laser of wavelength 633 nm and power level of 4.0 mW was used to excite the LRSPP mode in the input arm. The excitation setup is described in detail in [22]. A series of CCD images of the RI sensor in operation for different solutions with different refractive indices are shown in Figure 5. The CCD camera used was a DS-Ri1 with a sensitivity equivalent to ISO 200. The refractive index of the sample in the sample arm was varied by varying the sucrose concentration in deionized (DI) water, and the refractive index of the solution calculated using the details in [24].

**Figure 6:**
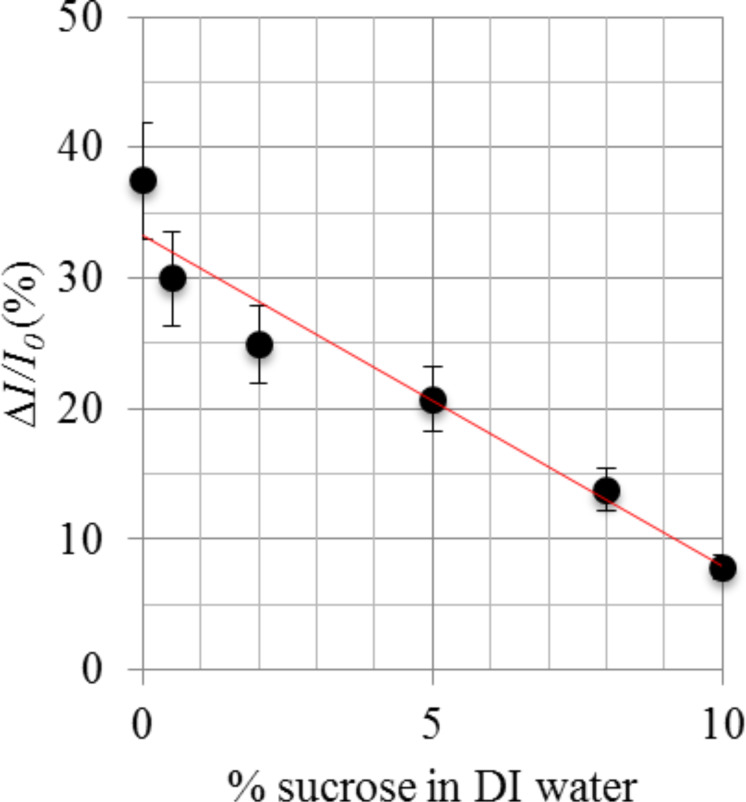
Relative intensity difference between the sample arm and reference arm versus weight percentage of sucrose in DI water. The error bars are for the standard error.

Maximum incoupling was ensured by adjusting the beam position on the input grating such that it obtains maximum intensity. Incoupling and outcoupling intensities of the sensor were analysed using CCD images. The outcoupling intensity of the reference and sample arm were measured for each sucrose solution. Figure 6 depicts the relative intensity change Δ*I*/*I*_0_ between the sample and reference arm versus sucrose weight percentage in each sucrose solution, where *I*_0_ is the intensity on the reference arm, *I*_r_ (Equation 3).

[6]
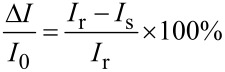


*I*_s_ is the sample arm output intensity at a specific sucrose concentration (Equation 5). *I*_0_ = *I*_r_ is the reference arm output intensity and the reference arm was covered by PMMA of refractive index 1.4888 (at 633 nm, 20 °C) [24]. Five RI sensors are used to calculate the relative intensity change for each sucrose measurements (using Equation 6), and the results averaged. The error bars in Figure 6 are for the standard error in this averaging.

**Figure 7:**
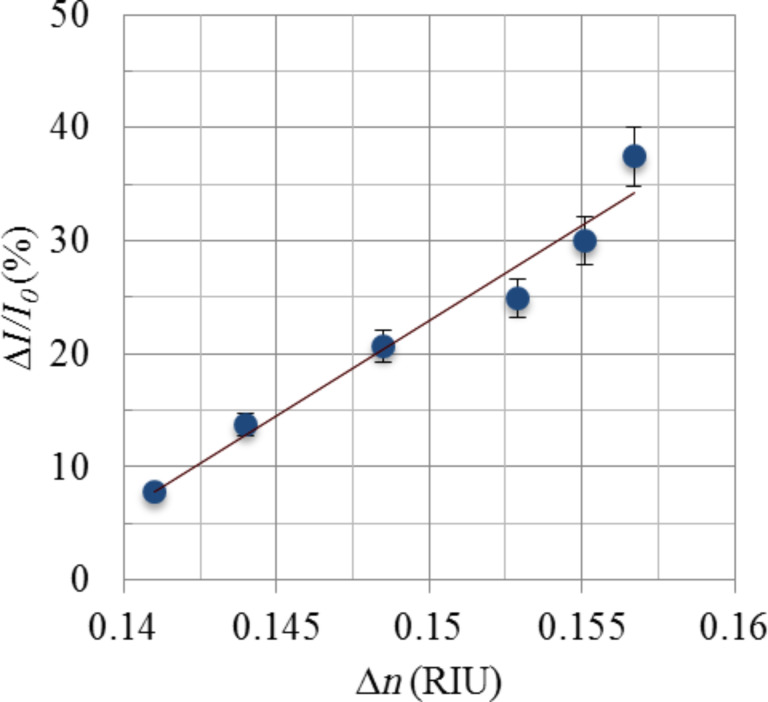
Relative intensity difference between sample arm and reference arm vs refractive index difference (between PMMA and sucrose solutions).

The relative intensity difference was highest for DI water (with no sucrose) and lowest for 10% sucrose in DI water. For further clarification, a graph was plotted against relative intensity change as a function of refractive index change with respect to PMMA (Figure 7). All the refractive indices of all sucrose solutions were measured at 20 °C at a wavelength of 633 nm relative to air [24].

We observed that the intensity difference between arms changes linearly with the change in the refractive index of the solution (w.r.t. PMMA). Figure of Merit (FOM) relative to the intensity change was calculated by dividing the difference in the relative intensity change ∆*I*/*I*_0_ by ∆*n*.

[7]
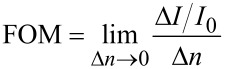


The calculated FOM for the relative intensity change of the device was ≈10^3^%/RIU. The gradient of the graph ∆*I*/*I*_0_ vs ∆*n* depicts the relative intensity difference for a refractive index change of 1 RIU in the sample arm. The device has a resolution of 6 × 10^−4^ RIU for a change in relative output intensity of 1%.

The sensor was further tested for 80% sucrose (R.I. 1.4906) and an index matching oil of RI 1.5150. For 80% sucrose solution and for index matching oil, the sample arm intensity was higher than the reference arm (refer to CCD images on Figure 5c and Figure 5d).

## Conclusion

We experimentally realised a three arm refractive index sensor design with a resolution of ≈6 × 10^−4^ RIU for a 1% relative intensity change between output arms. The device is highly compact and authors believe it’s the first attempt to fabricate an RI sensor with stripe waveguide coupling system.

## Supporting Information

File 1 More details for the total field of the coupled system.
