# Supplementary material for: Highly compact refractive index sensor based on stripe waveguides for lab-on-a-chip sensing applications
Source: Beilstein J Nanotechnol. 2016 May 25;7:751–7. doi: 10.3762/bjnano.7.66 (PMC4901982; doi:10.3762/bjnano.7.66)
Supplement: File 1 — More details for the total field of the coupled system. [file Beilstein_J_Nanotechnol-07-751-s001.pdf]

Supporting Information

for

Highly compact refractive index sensor based on stripe waveguides for lab-on-a-chip sensing applications

Chamanei Perera[1], Kristy Vernon*[1], Elliot Cheng[2], Juna Sathian[3], Esa Jaatinen[1] and Timothy Davis[4]

Address: [1]Science and Engineering Faculty, Queensland University of Technology, Brisbane 4001, Queensland, Australia, [2]Australian National Nanofabrication Facility QLD node, University of Queensland, St Lucia 4072, Queensland, Australia, [3]Department of Materials, Imperial College, London SW7 2AZ, UK and [4]School of Physics, University of Melbourne, Parkville 3010, Victoria, Australia

Email: Kristy Vernon - k.vernon@qut.edu.au

* Corresponding author

More details for the total field of the coupled system

A. Additional Theory information

Here we assume that propagating modes in all three stripes are identical and the amplitudes of each wave is represented by a_i (z) (input), a_r (z) (reference) and a_s (z) (sample) with direction of the propagating waves along z. Due to coupling between

the stripes, the amplitudes of the propagating modes decrease along the waveguides.

Changes in the amplitude of each wave on each arm as a function of z can be given by

$$\frac{da_i}{dz} = i\kappa(a_r + a_s) \quad \text{(S1)}$$

$$\frac{da_r}{dz} = i\kappa a_i \quad \text{(S2)}$$

$$\frac{da_s}{dz} = i\kappa a_i \quad \text{(S3)}$$

where κ is the coupling strength. The solution to the above equations is in the form of $e^{-i\gamma z}$ with γ satisfying

$$\gamma^3 - 2\kappa^2\gamma = 0 \quad \text{(S4)}$$

The solutions γ of the above equation (0, $\sqrt{2}\kappa, -\sqrt{2}\kappa$) are the wavenumbers of the amplitudes of each eigenmode in the coupled system. When γ = 0, the input arm has zero amplitude and the two outer arms have equal amplitudes but a π phase difference. This mode is depicted in Figure 2e and Figure 2f. When γ = $\sqrt{2}\kappa$, the mode exists in all three arms but the input arm is out of phase by π radians with the reference and sample arms. This mode is called the anti-symmetric mode and is shown in Figure 2c and Figure 2d The wave in the input guide starts interacting with the outer arms at $z = L_i$. a_r and a_s are zero at this point. During the coupling process the reference and sample arms are in phase with each other due to their identical parameters. The input LRSPP mode excites symmetric and anti-symmetric eigenmodes in the two outer arms (Figure 2a, Figure 2b and Figure 2c, Figure 2d) leading to an energy transfer from the input waveguide to the outer waveguides. Then the change in the amplitudes at $z = L_i$ are given by,

$$a_i(z) = A_i\left(e^{-i\gamma(z-L_i)} + e^{i\gamma(z-L_i)}\right) \quad \text{(S5)}$$

$$a_r(z) = \frac{1}{\sqrt{2}} A_i \left(-e^{-i\gamma(z-L_i)} + e^{i\gamma(z-L_i)} \right) \tag{S6}$$

$$a_s(z) = \frac{1}{\sqrt{2}} A_i \left(-e^{-i\gamma(z-L_i)} + e^{i\gamma(z-L_i)} \right) \tag{S7}$$

where A_i is the amplitude of the first (all arms are in phase) and second modes (reference and samples are π out of phase with the input arm) of the Figure 2a, Figure 2b and Figure 2c, Figure 2d respectively.

B. Choosing structural parameters

The chosen isolated stripes should support bound single modes. The coupling length L_c is defined as the distance the plasmon must travel before the full energy transfer occurs from the input waveguide to the two outer arms. This is dependent on the separation distance of outer the arms from the input waveguide (s) and the wavenumbers of the symmetric and anti-symmetric modes k_s and k_a (Ref. [19]).

$$L_c = \frac{\pi}{k_s - k_a} \tag{S8}$$

For the chosen s, L_c must be lower than the propagation distance of the plasmon mode and large enough to keep the reference and sample arms uncoupled to each other.

C. Additional Relative Intensity Difference Data

The 80% sucrose solution data was considered in this plot (Figure S1) but it was clear a linear fit no longer applied. This may be due to the 80% sucrose solution having a higher refractive index then the surrounding PMMA. Further work into sucrose solution’s with higher sucrose concentration needs to be performed in the future.

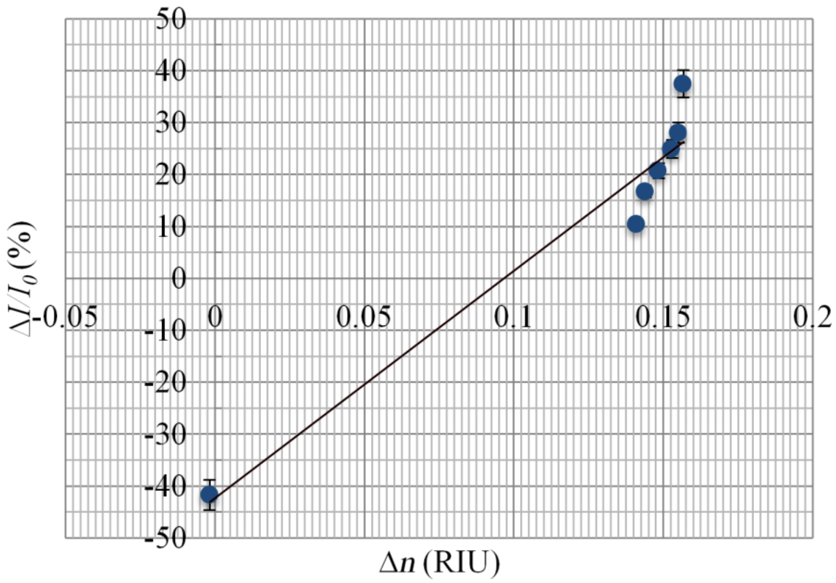

Figure S1: Relative intensity difference between the sample arm and reference arm versus weight percentage of sucrose in DI water. The error bars are for the standard error.
